# Chocolate and Prevention of Cardiovascular Disease: A Systematic Review

**DOI:** 10.1186/1743-7075-3-2

**Published:** 2006-01-03

**Authors:** Eric L Ding, Susan M Hutfless, Xin Ding, Saket Girotra

**Affiliations:** 1 Department of Epidemiology, Harvard University, School of Public Health, Boston, MA, USA; 2 Department of Nutrition, Harvard University, School of Public Health, Boston, MA, USA; 3 Department of Medicine, Medical College of Wisconsin, Milwaukee, WI, USA

## Abstract

**Background:**

Consumption of chocolate has been often hypothesized to reduce the risk of cardiovascular disease (CVD) due to chocolate's high levels of stearic acid and antioxidant flavonoids. However, debate still lingers regarding the true long term beneficial cardiovascular effects of chocolate overall.

**Methods:**

We reviewed English-language MEDLINE publications from 1966 through January 2005 for experimental, observational, and clinical studies of relations between cocoa, cacao, chocolate, stearic acid, flavonoids (including flavonols, flavanols, catechins, epicatechins, and procynadins) and the risk of cardiovascular disease (coronary heart disease (CHD), stroke). A total of 136 publications were selected based on relevance, and quality of design and methods. An updated meta-analysis of flavonoid intake and CHD mortality was also conducted.

**Results:**

The body of short-term randomized feeding trials suggests cocoa and chocolate may exert beneficial effects on cardiovascular risk via effects on lowering blood pressure, anti-inflammation, anti-platelet function, higher HDL, decreased LDL oxidation. Additionally, a large body of trials of stearic acid suggests it is indeed cholesterol-neutral. However, epidemiologic studies of serum and dietary stearic acid are inconclusive due to many methodologic limitations. Meanwhile, the large body of prospective studies of flavonoids suggests the flavonoid content of chocolate may reduce risk of cardiovascular mortality. Our updated meta-analysis indicates that intake of flavonoids may lower risk of CHD mortality, RR = 0.81 (95% CI: 0.71–0.92) comparing highest and lowest tertiles.

**Conclusion:**

Multiple lines of evidence from laboratory experiments and randomized trials suggest stearic acid may be neutral, while flavonoids are likely protective against CHD mortality. The highest priority now is to conduct larger randomized trials to definitively investigate the impact of chocolate consumption on long-term cardiovascular outcomes.

## Introduction

Cardiovascular disease (CVD), as a group, is a leading cause of the death in the United States [1], and worldwide, causing over 16.7 million deaths globally in 2002 [2]. In 1990, greater than 85,000,000 disability-adjusted life-years were lost worldwide due to coronary heart disease (CHD) and stroke; this CVD disease burden is projected to rise to 143,000,000 disability-adjusted life-years by 2020 [2]. Studies suggest cardiovascular diseases may be preventable by lifestyle modifications, such as exercise and nutrition [3-7]. Additionally, the American Heart Association, American Diabetes Association, and the U.S. Preventive Services Task Force have each indicated the likely importance of diet for the prevention of CVD [8-10].

In the American diet, fruits, vegetables, tea, wine and chocolate are major sources of antioxidants, which have been shown to have protective effects against CVD [11,12]. One class of antioxidants, flavonoids, commonly found in such foods, have attracted great interest in potentially lowering risk of CVD. Since cocoa products contain greater antioxidant capacity and greater amounts of flavonoids per serving than all teas and red wines [12,13], it is important to explore chocolate's potential effects on CVD.

Since ancient times, chocolate has long been used as a medicinal remedy [14] and been proposed in medicine today for preventing various chronic diseases [15,16]. While chocolate has also sometimes been criticized for its saturated fat content, mostly in the form of long-chain stearic acid, chocolate has also been lauded for its antioxidant potential. However, to this date there are no long-term randomized feeding trials of chocolate to assess effects on actual cardiovascular events. Nevertheless, there have been many short-term trials of cocoa and chocolate examining effects on cardiovascular intermediates, and numerous epidemiology studies of stearic acid and flavonoids exploring associations with cardiovascular outcomes.

This systematic review serves to comprehensively evaluate the experimental and epidemiologic evidence of cocoa and chocolate products. Particularly, we focus on the controversial potential benefits of the chocolate components stearic acid and flavonoids; review their overall effects on CVD risk factor intermediates and CVD endpoints; and conduct a meta-analysis of total flavonoid intake and risk of CHD mortality.

## Methods

We reviewed English-language MEDLINE publications from January 1965 through June 2005 for experimental, observational, and clinical studies of relations between the exposure search terms of chocolate, stearic acid, flavonoids (including flavonols, flavanols, catechins, epicatechins, and procynadins) and the outcome search terms of cardiovascular disease (coronary heart disease, ischemic heart disease, stroke), cholesterol, blood pressure, platelet, oxidation, and thrombosis. Approximately 400 papers were reviewed. Based on the relevance, strength, and quality of the design and methods, 136 publications were selected for inclusion.

We mainly focused on studies in humans, particularly randomized trials of either parallel or cross-over design, and prospective observational studies. Since no randomized trials have yet assessed chocolate in relation to definitive CVD outcomes, prospective observational studies evaluating chocolate sub-components and the risk of CVD outcomes were weighted equally in the overall evaluation. For overall objective evaluation, the strength of the evidence was evaluated by the design and quality of individual studies, the consistency of findings across studies, and the biologic plausibility of possible mechanisms. Finally, consistent with methods of the outdated prior analysis [17], an updated meta-analysis was conducted and relative risks estimates pooled using a random-effects model [18].

## Review

### Stearic acid in chocolate

Saturated fat has long been thought to contribute to atherosclerosis, and thus, adverse for CVD risk. However, stearic acid has been suggested to be a non-atherogenic type of dietary saturated fat. Stearic acid is a long-chain 18:0 saturated fatty acid found commonly in meats and dairy products. Cocoa butter, a fat derived from cocoa plants and predominantly found in dark chocolate [19], contains an average of 33% oleic acid (cis-18:1 monounsaturated), 25% palmitic acid (16:0 saturated), and 33% of stearic acid [20]. Thought it is generally considered that saturated fats overall adversely increase the total cholesterol and LDL levels [21-23], early studies have also suggested stearic acid may be non-cholesterolemic [21,22]. This has been confirmed in a series of studies and a meta-analysis of 60 controlled feeding trials which concludes stearic acid neither lowers HDL, nor increases LDL or total cholesterol [24-28]. The meta-analysis also estimates, that per 1% energy isocaloric replacement of stearic acid for carbohydrates, stearic acid intake is predicted to beneficially lower serum triglycerides by -17.0 nmol/L (p < 0.001) [26]. The most recent trial also shows the effects of stearic acid on lipids is even similar to oleic and linoleic acids [29].

Emerging studies have begun to explain how stearic acid in chocolate may be cholesterol-neutral. One suggested mechanism is stearic acid's lower absorption, which has been found in several animal and human studies [30-33], though only minimally in others [34,35]. These discrepancies may be attributed to the relative position of stearate on the triglyceride molecule which may affect its relative absorption rate [36,37]. This might also explain the suggestion that stearic acid from plants sources, such as cocoa, may be different from animal derived sources of stearic acid [38]. Furthermore, some feeding trials found lower absorption of cocoa buttered compared to corn oil [39], though not in others [40]. However, heterogeneity may be due to the dual-presence of calcium in chocolate, in which other trials found cocoa butter absorption further decreased 13% when supplemented with calcium (1% by weight) [41], as is done in chocolate manufacturing. Finally, another strongly supported protective mechanism relate to the relatively high percent desaturation of stearic acid to monosaturated oleic acid [35,42-45], a fat considered hypocholesterolemic [27,46-48] and protective against coronary heart disease [3,49].

Two other pathways suggested for potential benefit are stearic acid's potential anti-platelet and blood pressure reductions actions. Feeding trials have shown that stearic acid reduces mean platelet volume [50,51], an index of platelet activation. However, mixed findings have been observed regarding the relationship between stearic acids and factor VIIc coagulation factor, a predictor of fatal CHD [52-54]. Though an early study suggested that stearic acid may increase factor VIIc [55], no effect on levels of factor VIIc by stearic acid was observed in two other trials [56,57]. Moreover, additional trials have refuted the earlier small study and, in fact, shown that stearic acid lowered the levels of factor VIIc coagulation factor compared to palmitic [50,58] and other saturated fatty acids [58]. As for the relationship between stearic acid and blood pressure, two feeding trials found stearic acid did not adversely affect systolic blood pressure [28,59]. Furthermore, cross-sectional analysis within the Multiple Risk Factor Intervention Trial even found stearic acid levels may be inversely associated with diastolic blood pressure [60].

In summary, given the vast majority of studies showing stearic acid has beneficial or neutral effects on blood pressures and clotting parameters, it appears unlikely stearic acid intake would adversely affect CVD risk through these risk factors. Data indicates stearic acid does not adversely affect established traditional lipid risk factors, with even favorable lowering of serum triglycerides if isocalorically replaced for carbohydrates.

#### Stearic Acid Observational Studies

However, the observational studies of stearic acid's association with CVD are inconclusive. (Table 2) Among retrospective studies, a Japanese case-control study of serum levels reported no association for stenosis [61], a Norwegian study found lower odds of MI [62], while a Costa Rican study of dietary intake found higher risk of MI [63] with higher intake of stearic acid. However, the results from the Costa Rican study should not be given much weight since retrospective self-report of dietary intakes are notoriously inaccurate and susceptible to reporting bias [64]. Nevertheless, higher rates of CHD and CAD progression was found in several prospective studies [65-68], while stroke was not increased in another study [69].

**Table 1:** Summary of Chocolate and Cocoa Feeding Trials

**Author**	**Year**	**No. Participants**	**Trial Design**	**Duration**	**Intervention**	**Outcome(s)**
Kondo [83]	1996	12	Crossover	1 meal, pre/post-meal measurement	Cocoa (35 g delipidated), vs. none	Decreased LDL oxidation
Rein [138]	2000	30	Parallel	1 meal, 2 & 6 hrs	Cocoa beverage (300 ml, 19 g procyanidin), caffeinated beverage (17 mg caffeine), or water	Decreased platelet activation, decreased platelet function
Wang [79]	2000	20	Crossover	1 meal, 1 week/phase	Procyanidin-rich chocolate (27, 53, 80 g), vs. none	Increased antioxidant capacity, decreased oxidative stress
Osakabe [88]	2001	15	Parallel	daily, 2 weeks	Cocoa powder (36 g/day), vs. sugar	Decreased LDL oxidation (increased lag time)
Wan [85]	2001	23	Crossover	daily, 4 weeks/phase	Cocoa powder (22 g/day) + dark chocolate (12 g/day), vs. average American diet	Decreased LDL oxidation (increased lag time), Increased HDL concentration
Schramm [101]	2001	10	Crossover	1 meal, 2 & 6 hrs, 1 week/phase	Chocolate (35 g, high 4 mg/g vs. low 0.09 mg/g procyanidin)	Increased prostacyclin, decreased leukotriene (likely decreased platelet activation, anti-inflammatory)
Holt [95]	2002	18	Crossover	1 meal, 2 hrs	Chocolate chips (25 g semi-sweet), vs. none	Decrease platelet function
Mathur [86]	2002	25	Crossover	daily, 6 weeks/phase	Dark chocolate (37 g/day), cocoa powder (31 g/day), vs. none	Decreased LDL oxidizability, marginal HDL increase
Pearson [92]	2002	16	Crossover	1 meal, 1 day/phase	Cocoa beverage (300 ml, 19 g flavanol cocoa powder), cocoa beverage + aspirin, or aspirin	Decreased platelet activation, decreased platelet function, all additive of aspirin effects.
Heiss [99]	2003	20	Crossover	1 meal, 1 day/phase	Cocoa beverages (100 ml, high or low flavan-3-ol)	Increased NO bioactivity, improved endothelial function
Innes [97]	2003	30	Parallel	1 meal, 4 hrs	Dark (75% cocoa, highest flavonoid content), milk (20% cocoa), or white chocolate (no flavonoids)	Dark chocolate inhibited collagen-induced platelet aggregation
Murphy [94]	2003	32	Parallel	daily, 28 days	Cocoa flavonoid tablets (234 mg), vs. placebo	Decreased platelet function, no difference oxidation status
Serrafini [76]	2003	12	Crossover	1 meal, 1 day/phase	Dark chocolate (100 g), dark chocolate (100 g) + milk (200 ml), or 200 g milk chocolate	Increase antioxidant capacity, in absence of milk
Taubert [118]	2003	13	Crossover	daily, 14 days/phase	Dark chocolate (100 g, 500 mg polyphenols), vs. white chocolate (90 g, 0 mg polyphenols)	Lower systolic and diastolic blood pressure with dark chocolate
Wiswedel [90]	2004	20	Crossover	1 meal, 1 week washout	High flavanol (1.87 mg/ml) vs. low flavanol (0.14 mg/ml) cocoa beverage	Lower levels of lipid peroxidation indicators with high flavanol cocoa beverage
Engler [98]	2004	21	Parallel	daily, 2 weeks	Chocolate (high vs. low flavonoid)	Improved endothelial function, no difference oxidative stress, lipids with high flavonoid choc.
Mursu [115]	2004	45	Parallel	daily, 3 weeks	Dark chocolate, dark chocolate enriched with cocoa polyphenols, or white chocolate	Increased HDL concentration, no change LDL oxidizability
Grassi [116]	2005	15	Crossover	daily, 15 days/phase	Dark chocolate (100 g, 500 mg polyphenols), vs. white chocolate (90 g, 0 mg polyphenols)	Lower systolic blood pressure, improved insulin sensitivity, lower insulin resistance
Zhu [139]	2005	8	Parallel	1 meal, 1–2–4–8 hrs	Cocoa beverage (high flavonoid); 0.25, 0.38, 0.50 g/kg body weight dose	Reduced susceptibility to free-radical induced hemolysis
Vlachopoulos [140]	2005	17	Crossover	1 meal, 1 day/phase	Dark chocolate (100 g, 2.62 g procyanidin), vs. none	Improved endothelial function, vasodilation of brachial artery, no change in blood pressure
Fraga [119]	2005	28	Parallel	daily, 14 days	High flavanol milk chocolate (105 g, 168 mg flavanols) vs. low flavonoid chocolate (<5 mg flavanols)	Lower mean blood pressure, lower LDL cholesterol, lower oxidative stress markers in high flavanol chocolate group

**Table 2:** Observational Studies of Stearic Acid and Cardiovascular Outcomes

**Author**	**Year**	**Study design**	**N, Population**	**Stearic acid assessment method**	**CHD/MI Outcomes**	**Other**
Kromhout [141]	1995	Ecologic	12,763 men, 16 cohorts of 7CS	Dietary intake	↑ CHD mortality	
Simon [68, 69]	1995	Prospective	96 cases, 96 controls, USA-MRFIT	Serum levels	↑ CHD incidence	Null-stroke incidence
Watts [67]	1996	Prospective	50 men, Australia	Dietary intake	↑ CAD progression	
Hojo [61]	1998	Case-control	71 cases, 60 controls, Japan	Serum levels		Null-stenosis
Hu [65]	1999	Prospective	80,082 women, USA-nurses	Dietary intake	↑ CHD incidence	
Yli-Jama [62]	2002	Case-control	103 cases, 104 controls, Norway	Serum levels	↓ MI incidence	
Kabagambe [63]	2003	Case-control	485 cases, 508 controls, Costa Rica	Dietary intake	↑ MI incidence	
Wang [66]	2003	Prospective	3591 whites, USA	Serum levels	↑ CHD mortality	

Abbreviations: 7CS, 7 Countries Study; MRFIT, Multiple Risk Factor Intervention Trial;

* High stearic acid level among men from geographic areas of high IHD mortality

On the other hand, several limitations exist for observational studies of stearic acid. First, researchers have cautioned that analyses of dietary stearic acid are very difficult due to high correlations of stearic acid intake with other fatty acids (often r = 0.7 to 0.9), thus impeding optimal study of associations [65]. Additionally, the larger prospective study that found higher risk of CHD also noted chocolate was a very small contributor (5%) of total stearic acid intake, with red meats as primary sources of stearic acid. Finally, since there exists high interconversion of stearic acid to unsaturated fatty acids [35,42-45], studies involving serum levels of stearic acid do not answer the relevant causal question of dietary intake of stearic acid and risk of disease. The associations of long-term serum stearic acid levels represent the effects of post-conversion stearic acid levels after a large proportion of the original dietary stearic acid has already been converted away to monounsaturated fat, which is well-established to exert protective effects against CVD [3,27,46-49].

Thus, relatively little information can be inferred from observational studies of the association of stearic acid and CHD, and no epidemiologic study has, thus far, appropriately and optimally answered the causal question of the association of dietary stearic acid intake and risk of CVD. However, a sufficient body of strong evidence from short term randomized trials suggests stearic acid components in chocolate may be beneficial for cardiovascular health. However, further research in this area is warranted.

### Flavonoids in chocolate

**Figure 1:**
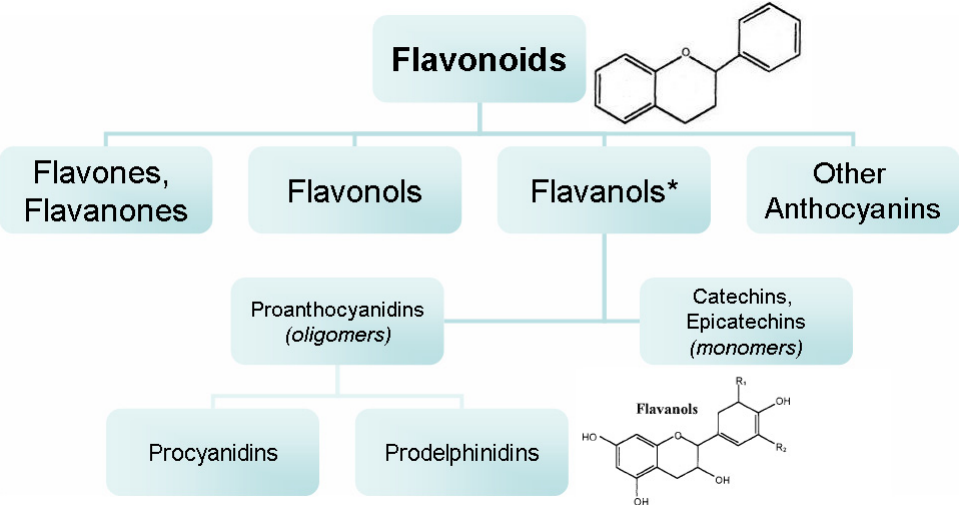
**Structural skeleton of flavonoids and classification hierarchy of common flavonoids**. *Flavanol is the predominate class of flavonoid found in cocoa and chocolate.

A 100 g bar of milk chocolate contains 170 mg of flavonoid antioxidants, procyanidins and flavanols [12]. It is estimated that chocolate is a leading source of procyanidin intake in Western nations (18–20%) [70,71]. Flavonoids belong to a class of antioxidants called polyphenols from plants [72]. The basic structure of flavonoids is a C6-C3-C6 backbone with two armomatic rings and varying degrees of hydroxylation differentiating one flavonoid type from another [73]. Flavonoids can be divided into various subclasses, important of which are flavones, flavonols, flavanones, catechins, anthocyanidins and isoflavones. Cocoa, is particularly rich in the flavonoids, epicatechin, catechin, and procyanidins (polymers of catechins and epicatechins) [74]. (Figure 1)

**Figure 2:**
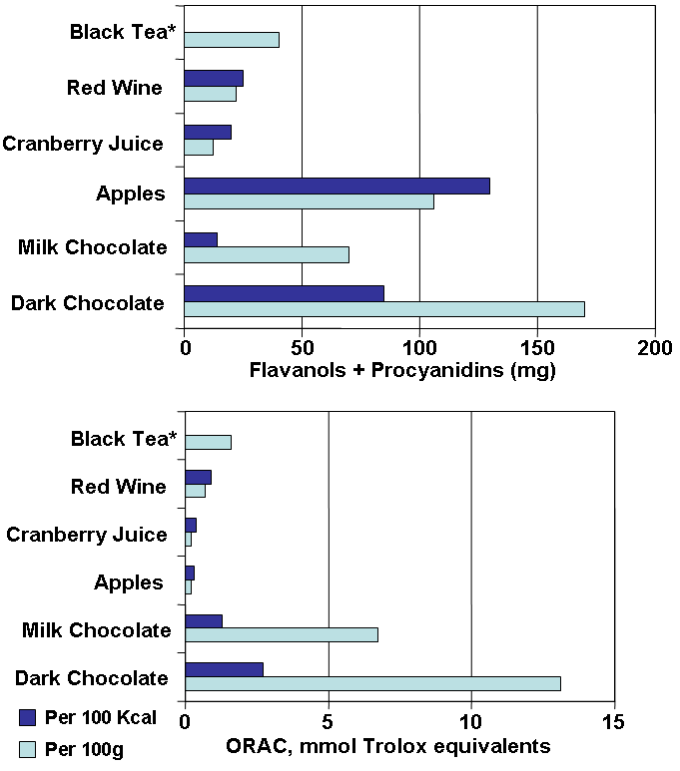
**Flavonoid content and antioxidant capacity (ORAC) of milk chocolate and dark chocolate versus other high flavonoid foods**. * Brewed, per 2 g bag/200 ml water. Antioxidant activity is reported as oxygen radical absorbance capacity (ORAC). Adapted from: Steinberg et al. J Am Diet Assoc 103: 215-23.

Various studies have compared the content of the flavanoids in cocoa with other food stuffs quantitatively. Figure 2 shows the comparative content of flavonoids in milk chocolate and dark chocolate versus other high-flavonoid foods. Cocoa has been shown to have the highest content of polyphenols (611 mg/serving) and flavanoids (564 mg/serving of epicatechin), greater than even tea and wine [13]. Per serving, dark chocolate contains substantially higher amounts of flavonoids than milk chocolate (951 mg of catechins per 40 g serving compared to 394 mg in white chocolate) [75], and levels of epicatechin in dark chocolate is comparable to red wine and tea [75]. Also of note, dark chocolate contains significantly greater amounts of total phenols as well as catechins than milk chocolate per serving (126+-7.4 μmol/g vs. 52.2+-20.2 μmol/g) [75]. In addition to dark chocolate having higher flavonoid content, the biologic effects of flavonoids may also be greater in dark chocolate because milk in milk chocolate may inhibit the intestinal absorption of flavanoids [76]. Finally, chocolate is also abundant in procyanidin flavonoids, comparable with levels in procyanidin-rich apples [77]. Thus, chocolate is a rich source of flavonoids, particularly catechins, epicatechins and procyanidins.

#### Mechanisms

Chocolate flavonoids have shown good dose-response bioavailability in humans [11,78,79]. There exists several mechanisms of how flavonoids may be protective against CVD; these include: antioxidant, anti-platelet, anti-inflammatory effects, as well as possibly increasing HDL, lowering blood pressure, and improving endothelial function. The body of trials involving chocolate flavonoids is summarized in Table 1.

Central to the pathogenesis of atherosclerosis is the oxidation of low-density lipoprotein (LDL). The chemical structure of flavonoids gives the compound free radical scavenging ability, which means flavonoids may have antioxidant effects [80]. Various studies have confirmed the role of flavanoids as antioxidants in biological systems. Flavanoids in chocolate have been shown to exert potent antioxidant effects in vitro assays under artificial oxidative stress [13,81-84] as well increase antioxidant capacity as part of various chocolate feeding trials [79,85-89]. Additionally, because lipid soluble flavonoids may intercalate into the membranes of lipoprotein particles, studies have shown flavonoids to decrease lipid peroxidation of biological membranes [90]. Furthermore, a randomized trial also demonstrated that flavonoid-rich foods can protect human lymphocytes from oxidative damage in vivo [91].

Additionally, aggregation of platelets at the site of plaque rupture and endothelial dysfunction has been implicated in the pathogenesis of atherosclerosis. Current research has shown that a number of components of chocolate, particularly catechin and epicatechin, have significant antiplatelet effects, quantitatively similar to that of aspirin [92]. Randomized trials studying platelet activation markers, microparticle formation and primary platelet aggregation as end points have found that daily intake of cocoa beverages produces a significant reduction in all these endpoints among healthy volunteers [93-96]. There were also significant correlations between the reduction in these end points and the plasma concentrations of catechin and epicatechin [93-96]. Another study found a significant reduction in platelet activation in groups consuming 100 g of dark chocolate when compared to those consuming similar amounts of white chocolate and milk chocolate [97]. In addition, randomized trials have also shown that consumption of high-flavanoid dark chocolate is associated with a significant improvement of endothelial function, marked by increase in brachial artery flow mediated dilation [98-100], likely mediated by chocolate flavonoids increasing local production of nitric oxide [99,100].

Chocolate may also influence levels of leukotrienes and prostacyclins. Leukotrienes are potent vasocontrictors, proinflammatory agents and stimulate platelet aggregation, whereas prostacyclin is a vasodilator and inhibits platelet aggregation. Consumption of chocolate with high procyanidin content (147 mg) was shown in a feeding trial to significantly lower the levels of leukotrienes (29%) and increase the levels of prostacyclin (32%) when compared to a group consuming a low procyanidin (3.3 mg) chocolate [101]. In vitro studies have indeed demonstrated chocolate components to inhibit lipoxygenase pathways, which gives rise to proinflammatory leukotrienes [102,103]. Inflammation is now recognized as another independent mechanism in the pathogenesis of atherosclerosis, with various inflammatory markers having been shown to predict risk of future CVD events [104-108]. In addition to anti-inflammatory effects on the lipoxygenase pathway, cocoa polyphenols have also been shown to decrease inflammation via several mechanisms, namely: inhibition of mitogen induced activation of T cells, polyclonal activation of B cells, reduced expression of interleukin-2 (IL-2) messenger RNA, and reduced secretion of IL-2 by T cells[109] Other have also found chocolate procyanidins can modulate of a variety of other cytokines (e.g. IL-5, TNF-α, TGF-β), reducing their inflammatory effects [110-114].

Furthermore, multiple cocoa feeding trials have also found chocolate to increase HDL cholesterol [85,86,115], and decrease blood pressure [116-119]. Finally, there are also suggestive findings in a few trials that indicate high-flavonoid chocolate may also lower LDL cholesterol [119], and improve insulin sensitivity [116].

Thus, the large body of evidence from laboratory findings and randomized trials suggest that high-flavonoid chocolate may protect against LDL oxidation, inhibit platelet aggregation, improve endothelial function, increase HDL, lower blood pressure, and reduce inflammation – thereby protective against risk of CVD.

#### Flavonoid Observational Studies

Mechanistic studies involving stearic acid and flavonoids have only assessed effects on intermediate cardiovascular endpoints. However, one cannot always assume effects from short term trials effects will necessarily translate into long term effects on CVD outcomes. Therefore, one needs to examine observational studies followed to CVD events. While one small study found moderate consumption of candy and chocolate was associated with lower all-cause mortality [120], this analysis neither isolates chocolate nor CVD events. Thus, in absence of specific studies of chocolate flavonoids and risk of CVD, studies of all flavonoids are the best available evidence to infer risk.

The prospective studies of flavonoids and risk of CVD are summarized in Table 3. The earliest international ecologic study suggested flavonoid intake may be associated with lower rates of CHD mortality [121]. While some studies report flavonoid intake is not associated with CHD incidence [122-124], two other prospective studies suggested flavonoids may lower risk of MI [125,126]. For stroke, the evidence is fairly consistent. Other than one small early study which found a significantly lower risk of stroke with higher total flavonoid intake [127], most studies indicated no association for risk of stroke [124,128-130]. However, most of these studies had insufficient power to adequately study stroke, nor enough power to stratify on various subtypes of stroke with different etiologies.

**Table 3:** Prospective Studies of Flavonoids and Cardiovascular Outcomes

**Author**	**Year**	**Study type**	**N, Population**	**Follow-up Years**	**Flavonoid Type**	**CHD/MI Incidence**	**CHD/MI Mortality**	**Stroke Mortality**	**Comments:**
Hertog [125, 142]*, Keli [127]	1993, 1996	Prospective	552 to 806 Men, Dutch	5, then 10*	Total Flavonoids	↓	↓	↓	*Update 1997 analysis finds even stronger CHD association [142]
Knekt [131]	1996	Prospective	5133 M+W, Finland	26	Total Flavonoids		↓		
Rimm [123]	1996	Prospective	34789 Men, USA	6	Total Flavonoids	Null	↓*		*marginal significance, if past history of CVD
Hertog [133]	1997	Prospective	1900 Men, UK	14	Total Flavonoids	Null	↑*		*marginal significance, *milk consumed w/tea
Yochum [130]	1999	Prospective	34492 PostM women, Iowa	10	Total Flavonoids		↓	Null	
Hirvonen [126, 129]	2000, 2001	Prospective	23596 Men, Finland	6.1	Total Flavonoids	↓ MI	↓*	Null	*suggestive, but non-significant
Arts [143]	2001	Prospective	806 men, Dutch	10	Catechins (Flavonoid)		↓	Null	
Arts [128]	2001	Prospective	34492 PostM women, Iowa	13	Catechins (Flavonoid)		↓		
Geleinjse [122]	2002	Prospective	4807 M+W, Dutch	5.6	Total Tea Flavonoids	Null	↓		
Knekt [132]	2002	Prospective	10054 M+W Finland	28	Specific flavonoids		↓	↓	also ↓ type 2 diabetes
Sesso [124]	2003	Prospective	38445 women, USA	6.9	Total Flavonoids	Null	Null	Null	
META-ANALYSIS (updated)**			**Total Flavonoids → CHD Mortality**			RR = 0.81 (95% CI: 0.71–0.92)*			**(extreme tertiles)**

**Updated meta-analysis includes: all studies of "total flavonoids" and CHD mortality; comparison of top vs. bottom tertile.

However, the most extensively consistent finding is the association between flavonoid intake and CHD mortality. A total of eight cohort studies found risk of lower CHD mortality with total or specific flavonoid intake [71,121,123,125,126,128,130-132], with one study finding marginally protective association among men with prior CVD conditions [123]. Only one study reported absolutely no association between flavonoid intake and CHD mortality [133]. However, as noted by the authors of one of the studies, a high background consumption of milk with tea intake may have led to the null finding [133], since milk intake has been shown to prevent the intestinal absorption of flavonoids [76].

A meta-analysis of the 7 prospective studies prior to September 2001 found that, overall, flavonoids may be protective against CHD mortality [17]. However, this meta-analysis did not include a large subsequent cohort study of 38,445 women [124], which found a non-significant inverse association between flavonoid intake and CHD mortality. However, results from our updated meta-analysis still indicate a significant protective association exists between flavonoid intake and risk of CHD mortality, RR = 0.81 (95% CI: 0.71–0.92), comparing highest vs. lowest tertiles.

However, a limitation of inference exists in that flavonoids consists of a wide variety of polyphenol compounds, the variety of which may differ between studies due to varying sources of dietary flavonoids. Nonetheless, dark chocolate does contain substantially more flavanols than tea, apple, onions, and red wine [12]. Additionally, chocolate has all the flavonoids of tea [134], has 4 times the catechins of tea [134], has many flavonoids not found in tea [135], and substantially contributes to the total flavonoid intake in the diet of many countries [136]. However, inference from observational studies on the protective effect of flavonoids in chocolate on CVD risk is somewhat indirect and may need to be examined by further studies.

Overall, these epidemiologic findings, combined with the large body of evidence from short term randomized chocolate feeding trials, suggests flavonoid intake from chocolate is likely protective against CVD, particularly CHD mortality. Additionally, given that dark chocolate has substantially higher levels of flavonoids than milk chocolate, and that milk may inhibit absorption of flavonoids – it would be more prudent to consume high flavonoid dark chocolate rather than milk chocolate.

## Conclusion

According to the International Cocoa Organization, production has risen from 1.2 million tons per year in 1960 to 3.2 million tons per year in 2004 [137]. Given the rapidly increasing world consumption of chocolate and rising global rates of CVD, it is important to establish chocolate's association with CVD risk. The projected increase in global consumption could have profound effects if chocolate consumption does have implications for CVD.

Based upon our systematic review, multiple lines of evidence from laboratory experiments and randomized trials suggest stearic acid may be neutral, while flavonoids are likely protective against CVD, the latter of which is well supported by prospective observational studies that suggest flavonoids may lower the risk of CHD mortality. Though it has been approximated that eating 50 g of dark chocolate per day may reduce one's risk of CVD by 10.5% (95% CI: 7.0%–13.5%) [16], such crude estimates were based on results from studies of short duration, extrapolated to long term CVD outcomes. Therefore, the highest priority now is to conduct long-term randomized feeding trials, beyond short term studies of CVD risk factor intermediates, in order to definitively investigate the impact of chocolate consumption on cardiovascular outcomes.

## Abbreviations

CHD, Coronary heart disease

CVD, Cardiovascular disease

CI, Confidence interval

HDL, High-density lipoprotein

IL, Interleukin

LDL, Low-density lipoprotein

NO, Nitric oxide

MI, Myocardial infarction

RR, Relative risk

## Competing interests

The author(s) declare that they have no competing interests.

## Authors' contributions

All authors contributed to systematically reviewing articles. E.L.D. led the drafting of the manuscript, insights into nutritional metabolism, and S.G. provided further insights into clinical disease etiology.
